# Epithelial JAM-A is fundamental for intestinal wound repair in vivo

**DOI:** 10.1172/jci.insight.158934

**Published:** 2022-09-08

**Authors:** Shuling Fan, Kevin Boerner, Chithra K. Muraleedharan, Asma Nusrat, Miguel Quiros, Charles A. Parkos

**Affiliations:** Department of Pathology, University of Michigan, Ann Arbor, Michigan, USA.

**Keywords:** Cell Biology, Gastroenterology, Inflammatory bowel disease

## Abstract

Junctional adhesion molecule-A (JAM-A) is expressed in several cell types, including epithelial and endothelial cells, as well as some leukocytes. In intestinal epithelial cells (IEC), JAM-A localizes to cell junctions and plays a role in regulating barrier function. In vitro studies with model cell lines have shown that JAM-A contributes to IEC migration; however, in vivo studies investigating the role of JAM-A in cell migration–dependent processes such as mucosal wound repair have not been performed. In this study, we developed an inducible intestinal epithelial–specific JAM-A–knockdown mouse model (*Jam-a^ERΔIEC^*). While acute induction of IEC-specific loss of JAM-A did not result in spontaneous colitis, such mice had significantly impaired mucosal healing after chemically induced colitis and after biopsy colonic wounding. In vitro primary cultures of JAM-A–deficient IEC demonstrated impaired migration in wound healing assays. Mechanistic studies revealed that JAM-A stabilizes formation of protein signaling complexes containing Rap1A/Talin/β1 integrin at focal adhesions of migrating IECs. Loss of JAM-A in primary IEC led to decreased Rap1A activity and protein levels of Talin and β1 integrin, and it led to a reduction in focal adhesion structures. These findings suggest that epithelial JAM-A plays a critical role in controlling mucosal repair in vivo through dynamic regulation of focal adhesions.

## Graphical abstract

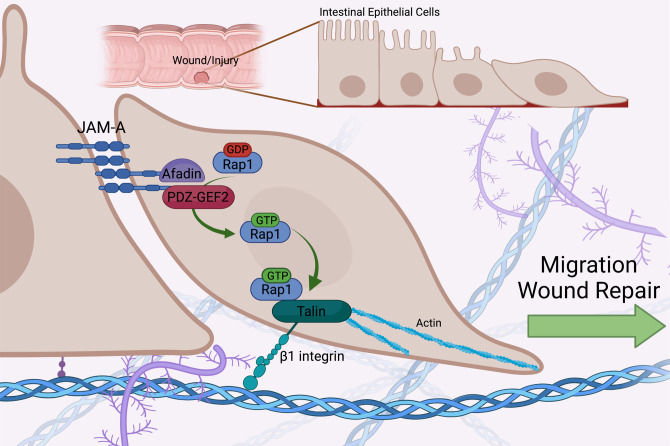

## Introduction

The intestinal mucosa is lined by a single layer of intestinal epithelial cells (IECs) that regulate paracellular transport of nutrients and water, while providing robust protection from external pathogens (1). In pathologic chronic inflammatory states such as ulcerative colitis (UC) and Crohn’s disease (CD), there is impaired healing of epithelial wounds, thereby causing these conditions to contribute to an exaggerated inflammatory response within the intestinal mucosa (2). Efficient repair of mucosal erosions and ulcers during active disease is, thus, critical to restore intestinal epithelial barrier (IEB) function and mucosal homeostasis (3).

In response to mucosal injury, IECs adjacent to wounds lose polarity, flatten, and migrate over erosions to restore barrier integrity. Such wound-associated epithelial cells undergo collective cell migration to initiate repair within a few hours of intestinal mucosal damage (1). Epithelial cell-cell junctions, as well as adhesive interactions with the substrate, play critical roles in regulating collective epithelial cell migration. In addition to regulating migration, epithelial cell-cell junctions, namely tight junctions (TJs), are composed of transmembrane proteins that interact with scaffolding proteins and signaling molecules that also play a critical role in regulating paracellular permeability and barrier integrity (4, 5).

An important TJ-associated transmembrane protein, junctional adhesion molecule-A (JAM-A), is a cortical thymocyte marker in xenopus (CTX) family member that has been shown to play a key role in intestinal homeostasis by regulating leukocyte trafficking and epithelial permeability (6). In vitro studies with model IEC have provided insights into mechanisms by which JAM-A functions relevant to findings in KO mice. Such studies indicate that JAM-A orchestrates the spatiotemporal association of scaffold molecules such as ZO-2 and Afadin that associate with Rap activators such as PDZ-GEFs to modulate activity of small GTPases in the Rap and Rho family, in order to regulate barrier function and integrin-dependent cell migration (7–9). These findings have provided important insights into how epithelial-expressed JAM-A regulates intestinal permeability. Such in vitro studies also predict that JAM-A expressed in the intestinal epithelium may play an important role in migration-dependent reparative processes. Despite these findings in model cell lines, in vivo studies probing the role of JAM-A in repair of mucosal wounds in the intestine have not been reported.

In this study, we show for the first time to our knowledge that mice with inducible loss of JAM-A in IECs and primary epithelial cell cultures (2D-colonoids) have profound defects in colonic mucosal wound repair in vivo and in vitro. With this murine model, we demonstrate that epithelial-expressed JAM-A regulates cell migration in a Rap1A/Talin/β1 integrin–dependent manner that leads to decreased formation of focal adhesion (FA) structures and impaired mucosal wound repair in vivo.

## Results

### JAM-A is required for colonic mucosal repair in vivo.

**Figure 1:**
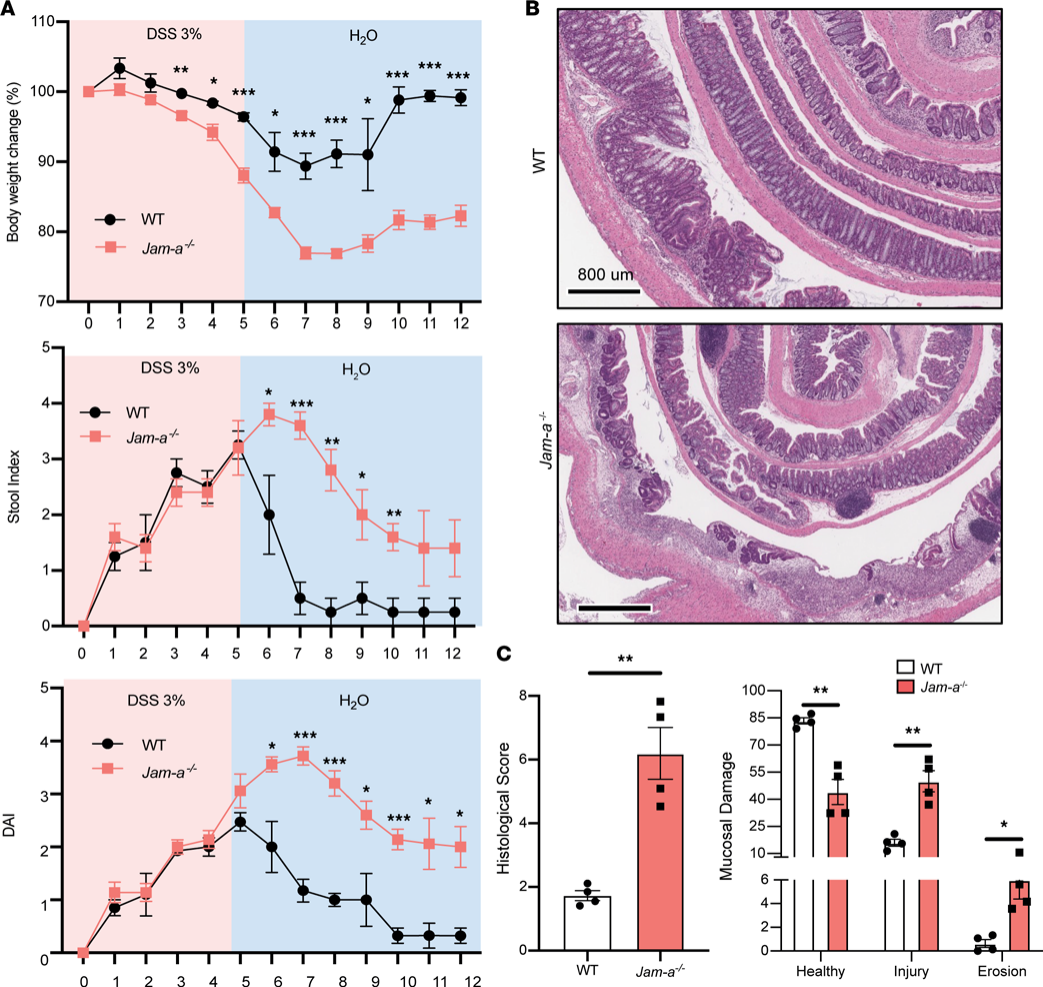
Mice with total loss of JAM-A have impaired recovery from DSS-induced mucosal injury in vivo. (**A**) Age- and sex-matched *Jam-a^–/–^* mice were treated with 3.0% DSS for 5 days, followed by 7 days of recovery. Following withdrawal of DSS after 5 days, *Jam-a^–/–^* mice exhibited greater body weight loss and a persistent increase of DAI during recovery compared with WT controls. JAM-A–deficient mice failed to recover the lost body weight compared with WT controls. (**B**) Representative H&E staining of colons harvested after 5 days of DSS treatment and 7 days of recovery revealed extensive mucosal injury, erosion, and ulceration in *Jam-a^–/–^* mice compared with WT controls. Scale bars: 800 μm. (**C**) Histological analysis of H&E-stained tissue sections of colon mucosa as seen in **B**. Histological colitis score (HCS) represents severity of mucosal injury based on the percentage of healthy versus, injured versus, eroded/ulcerated colon tissue of *Jam-a^–/–^* mice, and WT controls after recovery. After 7 days of recovery on drinking water, HCS in *Jam-a^–/–^* mice reveals greater mucosal damage in the absence of JAM-A. *Jam-a^–/–^* colon tissue contained more areas displaying signs of injury (such as damaged crypt architecture and immune cell infiltration) and eroded/ulcerated regions compared with control tissue. Total colon length was reduced in *Jam-a^–/–^* mice compared with WT mice. Dots represent individual mice. Data are representative of 2 independent experiments with 4 animals per group and are expressed as mean ± SEM. *P < 0.05, **P < 0.01, and ***P < 0.001 by 2-way ANOVA (**A** and **B**) and 2-tailed Student’s *t* test (**C**).

JAM-A has been shown to play an important role in the formation of epithelial cell-cell junctions. As the first transmembrane protein recruited to nascent TJs, JAM-A is critical to development of mature apical junctional complexes. Following injury, epithelial junctions are modified, and a complex remodeling process is required to loosen cell-cell contacts to facilitate cell migration. We previously reported that total loss of JAM-A in mice results in IEB compromise without spontaneous pathologic inflammation. However, these mice have increased severity of acute DSS-induced colitis characterized by increased mucosal ulceration, erosion, and injury (10). These observations suggest an increased susceptibility of the epithelium to injury and repair in JAM-A–deficient mice. To determine epithelial-specific contributions of JAM-A, since it is expressed in multiple cell types, more recent studies have analyzed the contribution of epithelial JAM-A in barrier defects. Indeed, selective loss of epithelial JAM-A in mice was shown to result in enhanced intestinal permeability analogous to the global JAM-A-KO (*Jam-a^–/–^*) mice (11). While such studies have shown that epithelial cell–specific JAM-A loss is clearly linked to intestinal barrier defects, no studies examining the contributions of JAM-A to mucosal epithelial responses to injury in vivo have been reported. Our initial studies examined mucosal repair in mice with global JAM-A loss in a model of dextran sodium sulfate–induced (DSS-induced) acute colonic mucosal injury followed by repair. After oral administration of 3% DSS (5 days), mice were given water to allow for recovery (7 days). In response to DSS treatment, *Jam-a^–/–^* and WT mice developed comparable clinical disease as assessed by disease activity index (DAI), calculated after analysis of body weight loss and fecal parameters including consistency and blood content (Figure 1A) (12). During water-only recovery, DAI was significantly worse in *Jam-a^–/–^* mice compared with that observed in WT controls, with 54% less weight recovery (Figure 1A). Histologic colitis scoring (HCS) of injured and eroded/ulcerated mucosa relative to normal confirmed significantly worse mucosal damage in *Jam-a^–/–^* mice compared with WT controls (Figure 1, B and C; 1.7 ± 0.2 versus 6.2 ± 0.8; *P* < 0.01). Comparison of percentages of normal, injured, and eroded/ulcerated mucosa in whole colon mounts showed that *Jam-a^–/–^* mice exhibited significantly increased mucosal injury damage (50% ± 5.8% versus 16.0% ± 1.9% control; *P* < 0.01), as well as more eroded/ulcerated regions in comparison with WT controls (6.0% ± 1.6% versus 0.6% ± 0.3%; *P* < 0.05) after 7 days of recovery (Figure 1C). These findings suggest that expression of JAM-A plays a critical role in the repair response to mucosal injury in the colon.

### Selective deletion of JAM-A in IECs impairs recovery from colitis.

**Figure 2:**
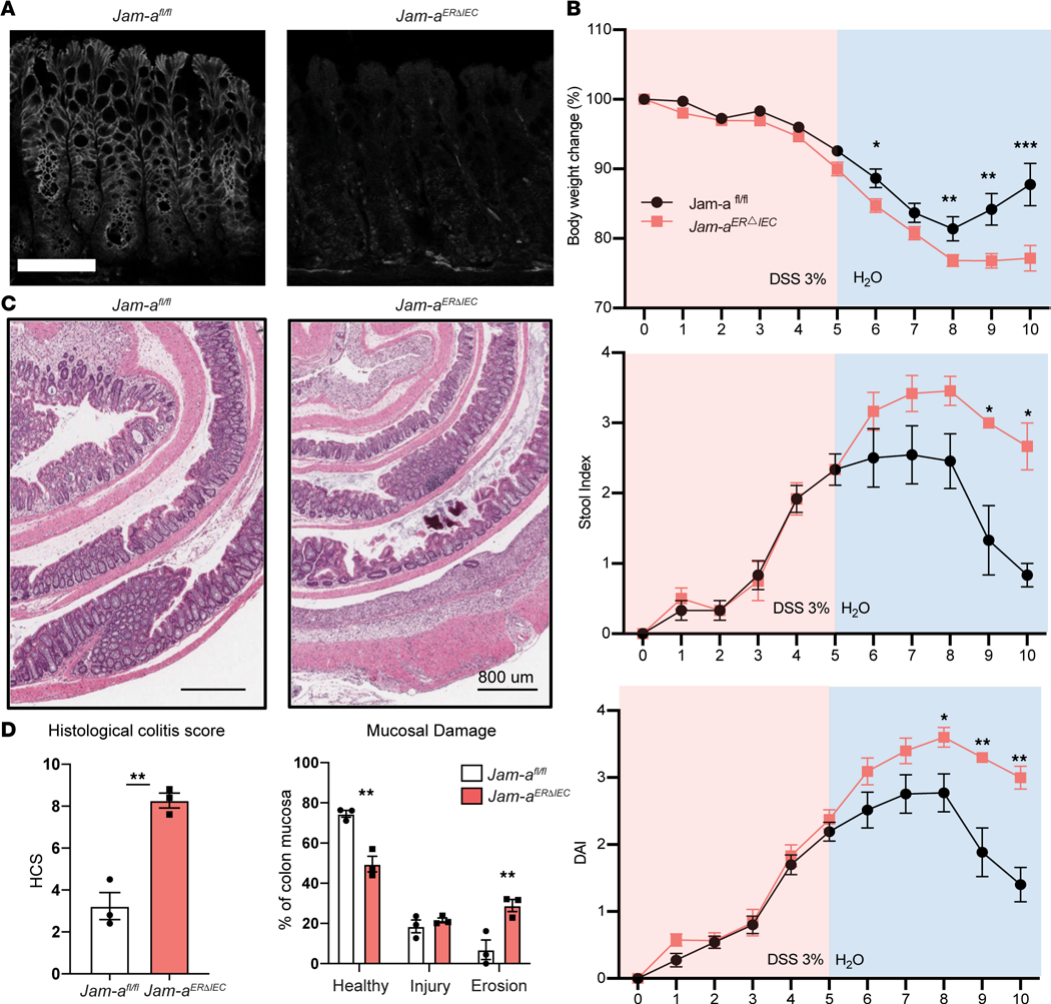
Epithelial expressed JAM-A is required for recovery from DSS-induced mucosal injury in vivo. (**A**) Naive and tamoxifen-treated *Jam-a^fl/fl^* and *Jam-a^ERΔIEC^* mice were analyzed for expression of *Jam-a* in IEC. Mice were treated with tamoxifen to induce acute depletion of JAM-A specifically in IEC and analyzed 30 days after treatment. Freshly frozen sections of colonic mucosa from tamoxifen-treated *Jam-a^fl/fl^* and *Jam-a^ERΔIEC^* mice were stained with anti–JAM-A antibody (white). Following tamoxifen treatment, JAM-A expression was observed to be depleted in IEC and preserved in lamina propria cells of *Jam-a^ERΔIEC^* mice. Scale bars: 100 μm. (**B**) After withdrawal of DSS, mice lacking epithelial JAM-A (*Jam-a^ERΔIEC^)* exhibited greater body weight loss and worse clinical scores (DAI) compared with controls. Data are representative of 2 independent experiments with 6 animals per group and are expressed as mean ± SEM. *P < 0.05, **P < 0.01, and ***P < 0.001 by 2-way ANOVA. (**C**) Representative H&E staining of colon tissue harvested after 5 days of recovery revealed extensive mucosal injury, erosion, and ulceration in *Jam-a^ERΔIEC^* mice compared with controls. Scale bars: 800 μm. Results are representative of 2 independent experiments with 3 animals per group. (**D**) Histological analysis of H&E-stained tissue sections of colon mucosa as seen in **C**. After 5 days of recovery on water, HCS in *Jam-a^ERΔIEC^* mice revealed greater mucosal damage following depletion of epithelial JAM-A. *Jam-a^ERΔIEC^* colon tissue contained more areas with mucosal injury and eroded/ulcerated regions compared with control tissue. Dots represent individual mice. Data are representative of 2 independent experiments with 3 animals per group and are expressed as mean ± SEM. *P < 0.05 by 2-tailed Student’s *t* test.

To specifically assess contributions of epithelial-expressed JAM-A on recovery from acute DSS-induced injury, we generated mice with targeted and inducible depletion of JAM-A in IECs. Epithelial-targeted *Jam-a^fl/fl^* mice were crossed with Villin Cre^ERT2^ (*Jam-a^ERΔIEC^*) (13). Acute loss of JAM-A in *Jam-a^ERΔIEC^* IECs was induced by tamoxifen (Tam) treatment for 15 days (14) and confirmed by immunofluorescence labeling of section of colonic mucosa and quantitative PCR (qPCR) (Figure 2A and Supplemental Figure 1; supplemental material available online with this article; https://doi.org/10.1172/jci.insight.158934DS1) of IEC. Under baseline conditions, colonic mucosa of Tam-treated JAM-A–deficient mice (*Jam-a^ERΔIEC^* + Tx) showed no gross histologic differences compared with Tam-treated controls (*Jam-a^fl/fl^* + Tx) (Supplemental Figure 1). Analogous to global JAM-A–KO mice, we noted similar DAI scores during induction of colitis but observed delayed recovery from DSS colitis in *Jam-a^ERΔIEC^* compared with control *Jam-a^fl/fl^* mice. *Jam-a^ERΔIEC^* mice gained 75% less body weight and exhibited more severe clinical symptoms (DAI 3.0) compared with control mice that had improved clinical symptoms (DAI 1.5) on day 10 during recovery from colitis. Consistent with DAI scores, histologic analyses of colons revealed significantly increased mucosal injury in mice lacking epithelial JAM-A compared with *Jam-a^fl/fl^* controls (Figure 2, B and C) on day 5 after DSS removal. Detailed histologic scoring confirmed significantly more mucosal damage in *Jam-a^ERΔIEC^* mice compared with controls (8.3% ± 0.3% versus 3.2 % ± 0.6%; *P* < 0.01). Percentages of normal, injured, and eroded/ulcerated regions of the mucosa in *Jam-a^ERΔIEC^* mice were similar those seen in total JAM-A KOs. Importantly, there was significantly more eroded/ulcerated mucosa when compared with controls after 7 days of recovery (28.9% ± 3.0% versus 6.9% ± 4.8%; *P* < 0.01) (Figure 2D). Collectively, these observations suggest that epithelial expression of JAM-A plays an important role in regulating intestinal mucosal repair in vivo.

### Epithelial JAM-A is required for repair of mucosal biopsy–induced injury in vivo.

**Figure 3:**
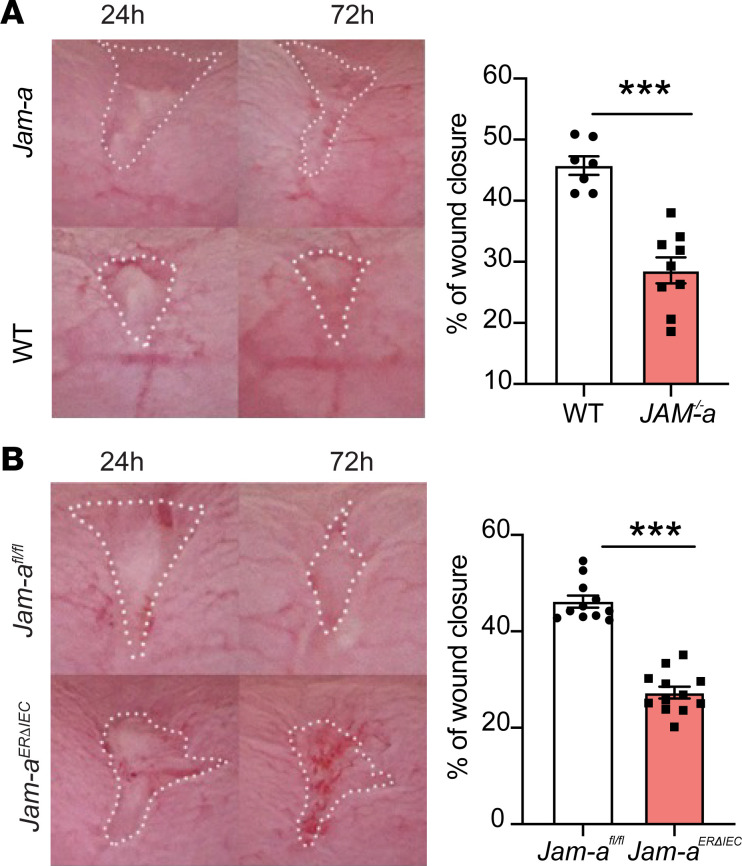
Epithelial JAM-A promotes biopsy-induced mucosal wound healing in vivo. Using a miniature endoscope and biopsy forceps, 4–5 mucosal wounds were generated along the mesenteric axis in the distal colon of anesthetized mice. (**A**) Analysis of wound surface area 24 and 72 hours after injury demonstrates a marked impairment of mucosal wound closure in *Jam-a^–/–^* mice. (**B**) Thirty days after tamoxifen-induced acute depletion of epithelial JAM-A, biopsy-based wounding was performed. Analysis of wound surface area after 24 and 72 hours revealed a significant reduction of wound closure in *Jam-a^ERΔIEC^* mice compared with littermate controls (*Jam-a^fl/fl^*). (**A** and **B**) Wound surface areas were traced manually (dotted lines) and measured digitally. Plotted dots represent mucosal wound closure of an individual animal, averaging wound closure of 4–5 wounds per mouse. Data are representative of 2 independent experiments with at least 3 mice per group (*Jam-a^–/–^* versus WT) or 5 mice per group (*Jam-a^ERΔIEC^* versus *Jam-a^fl/fl^*), respectively, and are presented as mean ± SEM. ***P < 0.001 by 2-tailed Student’s *t* test.

Given that *Jam-a^ERΔIEC^* mice had delayed recovery from colitis, we analyzed the contribution of epithelial JAM-A in mucosal repair using another in vivo model of colonic biopsy–induced injury. Wound repair in *Jam-a^–/–^* mice was compared with *Jam-a^ERΔIEC^* mice. Colonoscopy-based biopsy injury of the colonic mucosa was performed, and wound repair was examined by analysis of endoscopic video imaging. As shown in Figure 3, A and B, delayed mucosal wound healing was observed in both *Jam-a^–/–^* (32 2.8% *Jam-a^–/–^* versus 48 2.8% WT; *P* < 0.0001) and *Jam-a^ERΔIEC^* (27 1.8% *Jam-a^ERΔIEC^* versus 43 1.8% *Jam-a^fl/fl^*; *P* < 0.0001) mice when compared with respective controls. The wound healing defects observed in *Jam-a^ERΔIEC^* animals were similar to those observed in global *Jam-a^–/–^* mice, indicating that epithelial expression of JAM-A plays a major role in regulating intestinal mucosal wound repair in vivo.

### JAM-A regulates migration distance and velocity of primary IECs.

**Figure 4:**
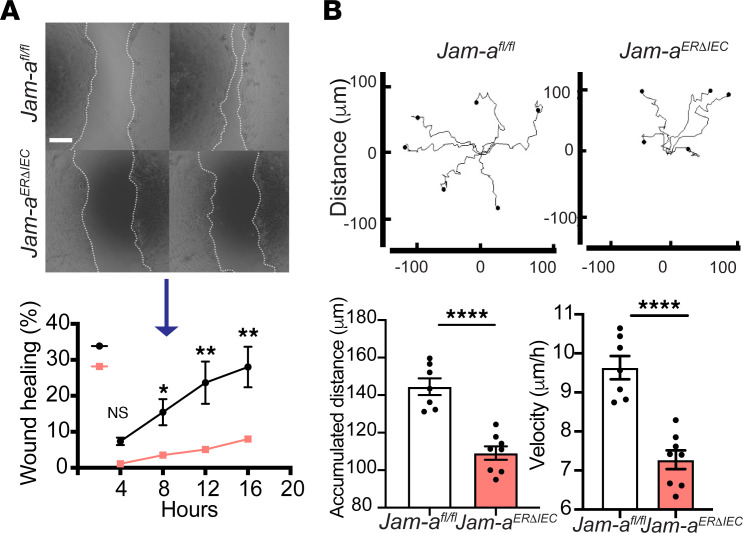
JAM-A regulates migration distance and velocity of primary intestinal epithelial cells. (**A**) pIEC monolayers derived from murine colonoids expressing JAM-A (*Jam-a^fl/fl^*) or acutely depleted of JAM-A (*Jam-a^ERΔIEC^*) were wounded and monitored for wound closure over 16 hours. Lack of JAM-A resulted in reduced wound closure at 8, 12, and 16 hours in *Jam-a^ERΔIEC^*–derived cells. Wound edges are highlighted by dotted lines. Scale bars: 50 μm. Data are representative of 3 independent experiments with 4 replicates per group and are represented as mean ± SEM. *P < 0.05, **P < 0.01 by 2-tailed, multiple-comparison *t* test. (**B**) Sparsely seeded primary intestinal epithelial cells were monitored via live-cell imaging over 16 hours to analyze nondirected migration. Dot plots display migration patterns observed in *Jam-a*^fl/fl^–derived — and *Jam-a^ERΔIEC^*–derived *—* colonoids, respectively. Each track represents an individual cell traced on leading fronts of each cell cluster. Loss of JAM-A resulted in a reduction of cell migration parameters, such as accumulated distance and velocity, in colonoids generated from *Jam-a^ERΔIEC^* when compared with controls. Dots represent individual cell clusters, based on the average of 4–5 individually traced cells per cluster. Data are representative of 3 independent experiments with at least 7 samples per group. Results are presented as mean ± SEM. ****P < 0.0001 by 2-tailed Student’s *t* test.

To verify in vivo findings and gain further insight into mechanisms governing intestinal epithelial–expressed JAM-A–directed wound repair, we generated monolayers of primary IECs (pIEC) derived from colons of *Jam-a^fl/fl^* and *Jam-a^ERΔIEC^* mice and performed scratch wound healing assays. Treatment of pIEC from *Jam-a^ERΔIEC^* with 1 μM Tam for 72-hour induced acute total depletion of JAM-A, as verified by immunoblotting and immunofluorescence labeling (Supplemental Figure 2, A and B) (15). Confluent pIEC monolayers were scratched, and wound closure was determined via time-lapse imaging over 16 hours using previously described methods (9). Significantly delayed wound closure was observed in *Jam-a^ERΔIEC^* pIEC beginning at 8 hours, with enhanced impairment at 12 and 16 hours after injury (*P* < 0.05, 8 hours; *P* < 0.01, 12 and 16 hours) (Figure 4A). These results were corroborated in studies with a human model IEC line, T84 cells lacking JAM-A after lentiviral transduction of JAM-A shRNA (JAM-A KD; Supplemental Figure 2C). Analogous to *Jam-a^ERΔIEC^* colonoids, we observed delayed repair of scratch wounds in SKCO-15 IEC after JAM-A knockdown (Supplemental Figure 2D).

Since wound repair is achieved by collective IEC migration, we analyzed contributions of JAM-A to cell migration by tracing and quantifying motility of leading-edge cells in low-density spreading colonoids. We observed that pIEC derived from *Jam-a^ERΔIEC^* mice traveled shorter distances and at lower velocities than *Jam-a^fl/fl^* control cells. *Jam-a^ERΔIEC^* colonoids adjoining wounds (leading edge cells) migrated 109 μm over 16 hours (accumulated distance) in comparison with WT pIEC that migrated 144 μm (109 ± 3.6 μm versus 144 ± 4.4 μm; *P* < 0.001), resulting in a 25% reduction in accumulated distance, as well as significantly decreased velocity (7.8 ± 0.2 μm/h *Jam-a^ERΔIEC^* versus 9.7 ± 0.3 μm/h *Jam-a^fl/fl^*; *P* < 0.001) (Figure 4B). These findings further support an important role of JAM-A in regulating intestinal epithelial wound healing by controlling velocity and distance of cell migration.

### JAM-A promotes FA formation in pIEC during cell migration and wound repair.

**Figure 5:**
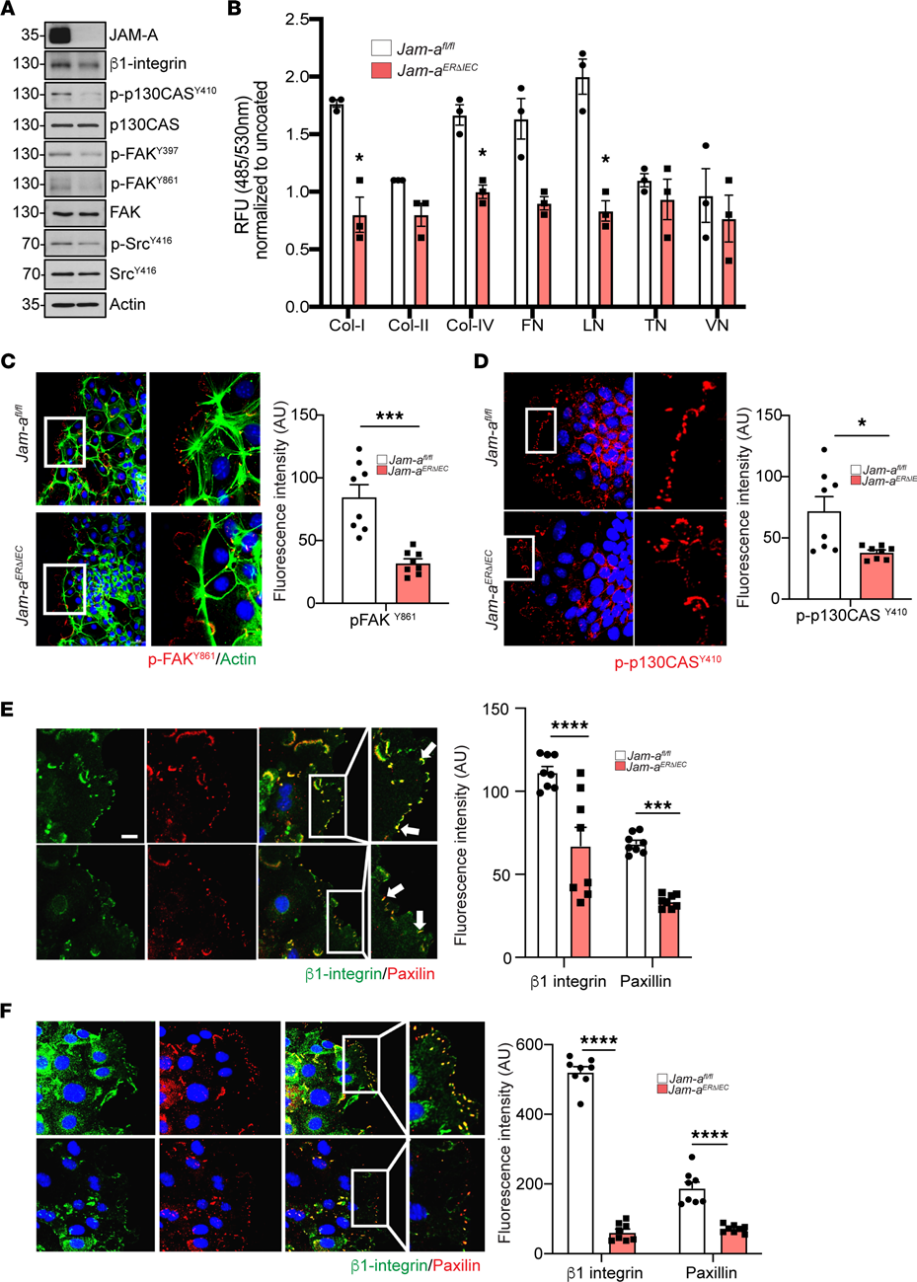
JAM-A promotes focal adhesion formation in primary IECs during cell migration and wound repair. pIEC were treated with tamoxifen prior to inducing acute loss of epithelial JAM-A in *Jam-a^ERΔIEC^-*derived cells. (**A**) Lysates of spreading pIEC derived from *Jam-a^fl/fl^* and *Jam-a^ERΔIEC^* mice were subjected to immunoblot for focal adhesion molecules known to be associated with regulation of β1 integrin-dependent cell adhesion. Loss of JAM-A revealed decreased β1 integrin protein expression and reduced phosphorylation of FAK^Y861^, p130CAS^Y410^, and paxillin^Y118^ in cells derived from *Jam-a^ERΔIEC^* mice. (**B**) Binding capacity of freshly isolated murine crypts from *Jam-a^fl/fl^* and *Jam-a^ERΔIEC^* to different extracellular matrixes. (**C**) Lamellipodia of migrating *Jam-a^ERΔIEC^*–derived pIEC exhibited fewer FAK^Y861^-positive focal adhesions and displayed less organized distribution of FAK^Y861^-positive focal adhesions when compared with *Jam-a^fl/fl^* controls. Scale bar: 50 μm. (**D**) Lamellipodia of migrating *Jam-a^ERΔIEC^–*derived pIEC demonstrated reduced number of p130Cas^Y410^-positive focal adhesions when compared with *Jam-a^fl/fl^* controls. Scale bars: 50 μm. (**E**) Confocal microscopy of lamellipodia of migrating primary epithelial cells generated from *Jam-a^ERΔIEC^* mice revealed reduced expression and disrupted colocalization of the associated focal adhesion proteins β1 integrin and paxillin compared with *Jam-a*^fl/fl^ cells. Scale bars: 25 μm. (**F**) pIEC generated from *Jam-a^fl/fl^* and *Jam-a^ERΔIEC^* mice were subjected to a scratch wound and imaged by confocal microscopy 6 hours after injury. pIEC immediately adjacent to the wound showed reduced expression and disrupted colocalization of the associated focal adhesion proteins β1 integrin and paxillin in *Jam-a^ERΔIEC^* cells compared with *Jam-a^fl/fl^* cells (inset). Scale bars: 25 μm. All results are representative of 3 independent experiments; data are expressed as mean ± SEM. *P < 0.05,***P < 0.001, ****P < 0.0001 by 2-way ANOVA (**B**) and 2-tailed Student’s *t* test (**C**–**F**). Col, collagen; FN, fibronectin; LN, laminin; TN, tenascin; VT, vitronectin.

Previous studies using cultures of model IECs demonstrated that JAM-A promotes cell migration through activation of the small GTPase Rap1 and stabilization of β1 integrin protein (9). To determine mechanisms regulating JAM-A–dependent cell migration in natural intestinal epithelium, we performed experiments using primary murine IEC deficient in JAM-A. It is well appreciated that, in migrating cells, β1 integrin regulates FA formation by facilitating recruitment and autophosphorylation of the FA-associated proteins FA Kinase (FAK) and Src, followed by the activation of paxillin and p130Cas by these kinases (16, 17). To study downstream signaling molecules of JAM-A–dependent β1 integrin–mediated cell migration, we analyzed FA-associated proteins in spreading and migrating pIEC cultures. Immunoblot analysis of collectively migrating *Jam-a^ERΔIEC^* pIEC showed a 35% decrease in β1 integrin protein expression compared with *Jam-a^fl/fl^* controls (0.99 ± 0.03 versus 0.65 ± 0.08; *P* < 0.05). In addition, there was decreased phosphorylation of FAK at tyrosine 397 (p-FAK^Y397^, 40% decrease; 1.17 ± 0.03 versus 0.67 ± 0.02; *P* < 0.001) tyrosine 861 (p-FAK^Y861^, 80% decrease; 0.97 ± 0.01 versus 0.2 ± 0.02; *P* < 0.001), p130Cas at tyrosine 410 (p-p130Cas^Y410^, 62% decrease; 1.00 ± 0.03 versus 0.38 ± 0.02; *P* < 0.001), and Src-kinase phosphorylation at tyrosine 416 (p-Src^Y416^, 30% reduction; 1.00 ± 0.03 versus 0.69 ± 0.08; *P* < 0.05) with no change in total FAK, Src, or p130Cas expression in *Jam-a^ERΔIEC^* pIEC compared with *Jam-a^fl/fl^* (Figure 5A). To further demonstrate a defect in cell matrix adhesion in *Jam-a^ERΔIEC^* pIEC, we performed a fluorumetric extracellular matrix cell adhesion array. We evaluated binding capacity of freshly isolated murine crypts from *Jam-a^fl/fl^* and *Jam-a^ERΔIEC^* to different extracellular matrixes: Collagen I, II, and IV; fibronectin; laminin; tenascin; and vitronectin. We found that pIEC cultures lacking JAM-A had a significant defect in binding to Collagen I and IV and to laminin (Figure 5B). Immunofluorescence labeling of p-FAK^861^ and p-p130Cas^Y410^ in *Jam-a^ERΔIEC^* pIEC plated in low density revealed reduced basal staining (p-FAK^861^, 84.9 ± 9.7 arbitrary units (au) versus 32.1 ± 3.3 au, *P* <0.001; p-p130Cas^Y410^, 72.1 ± 11.5 au versus 38.1 ± 1.9 au, *P* <0.05) and disrupted punctate structures reminiscent of FAs along leading edges of migrating cells when compared with control pIEC (Figure 5, C and D). Furthermore, there were significantly decreased levels of β1 integrin and paxillin at the leading edges of migrating pIEC derived from *Jam-a^ERΔIEC^* compared with controls (*Jam-a^fl/fl^*) (β1 integrin, 111.5 ± 3.5 au versus 67.2 ± 11.1 au, *P* < 0.0001; paxillin, 68.5 ± 2.1 au versus 33.7 ± 1.4 au, *P* < 0.001; Figure 5E). To further probe the mechanistic link between JAM-A and β1 integrin–mediated FA formation during wound repair, we analyzed β1 integrin and paxillin localization at the leading edge of scratched 2D enteroids monolayers 6 hours after injury. Confocal imaging revealed a marked decrease in labeling of both proteins in JAM-A–deficient monolayers (*Jam-a^ERΔIEC^*) compared with controls (*Jam-a^fl/fl^*) (β1 integrin, 521.4 ± 15.3 au versus 61.7 ± 8.9 au, *P* < 0.0001; paxillin, 188.5 ± 17.0 au versus 70.9 ± 3.8 au, *P* < 0.0001; Figure 5F). Collectively, these observations implicate an important role for JAM-A in regulation of β1 integrin–mediated FA formation and are consistent with JAM-A–dependent pathways that mediate β1 integrin function and regulate downstream signaling in FAs during cell migration and wound repair.

### JAM-A regulates Talin recruitment to FAs during cell migration and wound repair.

**Figure 6:**
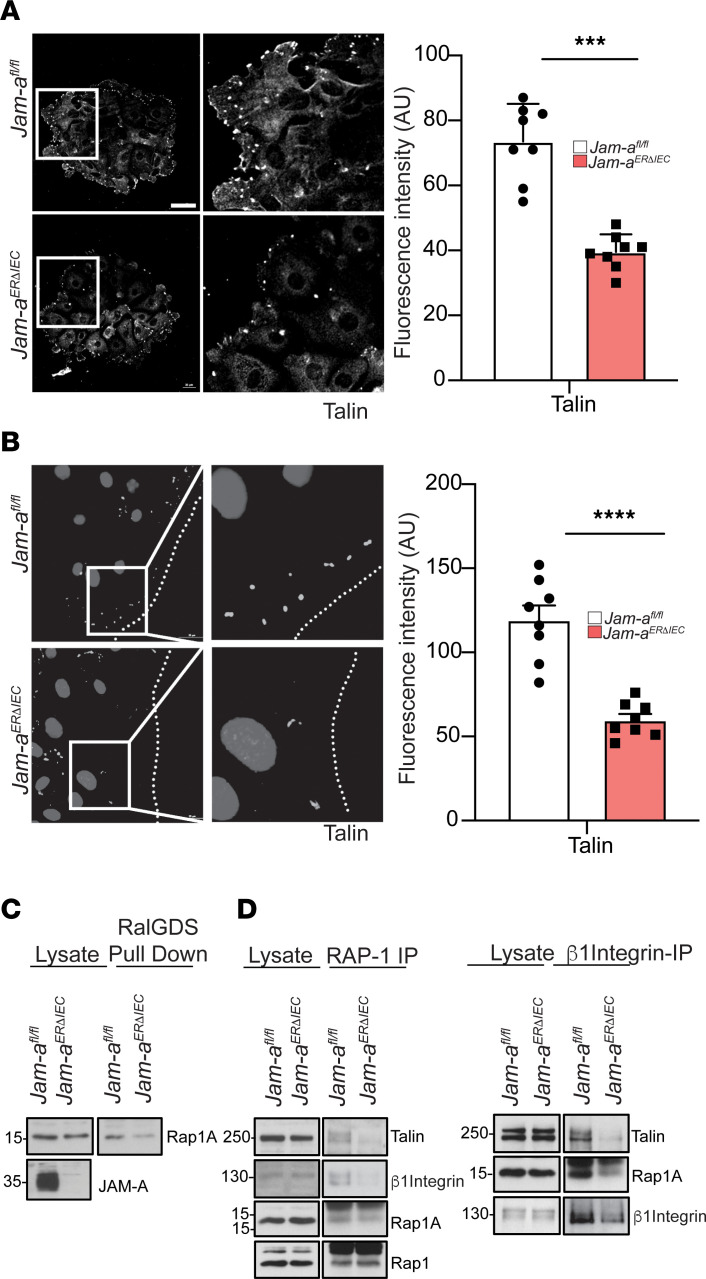
JAM-A regulates Talin recruitment to focal adhesions during cell migration and wound repair. pIEC generated from *Jam-a^fl/fl^* and *Jam-a^ERΔIEC^* mice were treated with tamoxifen prior to induce acute loss of epithelial JAM-A in *Jam-a^ERΔIEC^–*derived cells. (**A**) Spreading pIEC were imaged by confocal microscopy. Lamellipodia of migrating JAM-A–deficient cells (*Jam-a^ERΔIEC^*) exhibited lower numbers and disrupted distribution of Talin^+^ focal adhesions when compared with *Jam-a^fl/fl^* controls (inset). Scale bars: 50 μm. Results are representative of 3 independent experiments. (**B**) pIECs were subjected to a scratch wound and imaged by confocal microscopy 6 hours after injury. Imaging of cells generated from *Jam-a^ERΔIEC^* mice in leading edges revealed fewer Talin^+^ focal adhesions compared with *Jam-a^fl/fl^* controls (inset), with comparable numbers of Talin containing focal adhesions in “following” cells of JAM-A–deficient and JAM-A–expressing cells. Scale bars: 50 μm. Results are representative of 3 independent experiments. (**C**) Lysates from spread primary intestinal epithelial cells were incubated with RalGDS RBD beads to pull down Rap1A from *Jam-a^ERΔIEC^* and *Jam-a^fl/fl^* mice. (**D**) Lysates from spread primary IECs with JAM-A deficiency or floxed controls were immunoprecipitated with anti-Rap1A (left panel) or anti–β1 integrin (right panel) antibodies followed by immunoblotting with anti-talin, β1 integrin, and Rap1A antibodies to evaluate the presence of a functional protein complex containing Rap1A/talin/β1 integrin. IECs from *Jam-a^ERΔIEC^* mice are less able to form a Rap1A/talin/β1 integrin complex compared with IEC from control *Jam-a^fl/fl^* mice.

The cytosolic protein Talin bridges the actin cytoskeleton and integrins, and Talin binding to the β-subunit of integrins is required for β1 integrin activation and cell spreading (18). We hypothesized that JAM-A may promote β1 integrin–mediated cell migration by regulating Talin recruitment through Rap1. Immunofluorescence labeling of Talin revealed a significant decrease in Talin at the leading edge of spreading subconfluent JAM-A–deficient enteroids (*Jam-a^ERΔIEC^*) compared with controls (73.5 ± 4.1 au versus 39.5 ± 1.9 au; *P* < 0.001; Figure 6A). Furthermore, similar results were obtained at the leading edge of enteroids migrating to heal scratch wounds (119.4 ± 8.5 versus 59.9 ± 3.6; *P* < 0.0001; Figure 6B). These findings suggest that JAM-A regulates Talin recruitment to FA at the leading edge of migrating cells during wound repair. Since Talin interacts with Rap1A/1B and promotes its binding to and activation of β integrins, we examined whether Rap1 activity is altered in pIEC lacking JAM-A expression. A RalGDS (a GEF protein that interacts only with active Rap1) pulldown assay revealed decreased active Rap1 in spreading and migrating cells in subconfluent JAM-A–deficient IECs (Figure 6C and Supplemental Figure 3). To gain insight into how JAM-A may regulate Talin recruitment and β1 integrin activity during cell migration, we examined whether Rap1 is directly associated with either Talin or β1 integrin in pIEC. Co-IP assays with Rap1 and β1 integrin in migrating pIEC of control *Jam-a^fl/fl^* and *Jam-a^ERΔIEC^* confirmed that Talin, β1 integrin, and Rap1 form a JAM-A–dependent complex (Figure 6D). IP of Rac1A and β1 integrin demonstrated decreased Co-IP levels of Talin in *Jam-a^ERΔIEC^* pIEC when compared with *Jam-a^fl/fl^*. Furthermore, lower levels of Rap1A were observed in β1 integrin immunoprecipitates, as well as β1 integrin in Rap1A immunoprecipitates (Figure 6D). Similar results were obtained in Co-IP experiments using human SKCO-15 model IEC after JAM-A knockdown compared with controls (Supplemental Figure 4). Collectively, these data suggest that JAM-A plays an important role in regulating Talin recruitment to FAs during IEC migration and wound healing. Furthermore, these findings suggest that JAM-A mediates β1 integrin–dependent epithelial cell migration by regulating the association and formation of a Rap1/Talin/β1 integrin complex in FAs of migrating pIEC.

## Discussion

JAM-A contribution to IEB function has been demonstrated. Mice with global JAM-A KO have increased intestinal epithelial permeability and epithelial proliferation compared with controls (10, 19). Additionally, in vitro studies suggest that loss of JAM-A resulted in decreased IEC migration that was associated with decreased levels of β1 integrin (9). Nevertheless, reports in several cancer cell types — such as breast and renal — identified increased epithelial migration in vitro after JAM-A knockdown, which is the opposite effect of IECs (20–23). Such observations suggest that JAM-A may activate distinct signaling pathways in different tissues or/and cell types during epithelial cell migration. In contrast, we have reported decreased IEC migration after expression of mutant JAM-A proteins that are defective in dimerization or lacking the PDZ binding motif (24).

A limitation of previous in vivo studies aimed at understanding JAM-A function has been the use of total JAM-A–KO mice, which lack the ability to distinguish between the relative contributions of JAM-A–deficient cell types, making it difficult to dissect specific roles of epithelial expressed JAM-A in different physiological and pathological processes.

Here, we provide potentially new in vivo and in vitro mechanistic insights into the function of intestinal epithelial expressed JAM-A in response to chemical (DSS) or mechanical induced wounds. We show that epithelial JAM-A expression is essential for intestinal mucosal wound healing, as Tam-treated *Jam-a^ERΔIEC^* mice exhibited significant wound healing defects after biopsy-induced injury, as well as impaired mucosal healing after DSS induced colitis.

In a previous report, we observed enhanced disease during acute DSS (5%) treatment in JAM-A–KO mice; however, these differences were noted in an experimental model, where 5% DSS was continuously administrated for 7 days followed by euthanasia (10). In this study, experiments were designed to examine recovery after acute administration of a lower concentration of DSS (3%) over a shorter period (5 days). Using this shortened DSS treatment protocol, there were no significant differences in the DAI between JAM-A–KO or JAM-A^ERΔIEC^ mice compared with controls. However, results of these in vivo experiments conclusively demonstrate the importance of epithelial expressed JAM-A in facilitating colonic mucosal repair. To directly quantify mucosal injury from DSS experiments, we utilized a histological colitis score (HCS) to evaluate prevalence of erosion and ulceration along the entire length of the colon (12). HCS analysis confirmed that JAM-A–KO and JAM-A^ERΔIEC^ mice develop more severe colitis than respective controls, as shown by increased evidence of mucosal injury and epithelial erosion. We confirmed the JAM-A–dependent mucosal repair defect independently with a colonoscopy-based biopsy induced colonic injury model. Both, JAM-A–KO and JAM-A^ERΔIEC^ mice displayed significantly reduced colonic mucosal healing compared with controls. Interestingly, we previously found that intestinal mucosal samples from people with inflammatory bowel disease and mice with DSS-colitis showed JAM-A phosphorylation at tyrosine 280, while control human and murine mucosa did not. It was determined that tyrosine phosphorylation promotes JAM-A internalization and mimicking total loss of JAM-A, corroborating the importance of JAM-A expression in maintenance of gut homeostasis (25). Collectively, results from these experiments demonstrate that JAM-A is essential for mucosal repair/recovery in the intestine in vivo.

Repair of wounds in colonic mucosa requires the collective migration and proliferation of IECs to cover erosions and ulcers. Efforts were focused on better understanding how JAM-A regulates IEC migration to gain mechanistic insights behind the pronounced wound repair defects observed in JAM-A–KO and JAM-A^ERΔIEC^ mice. During repair of mucosal erosions, a single layer of nonproliferative highly migratory epithelial cells known as wound-associated epithelium (WAE) emerge from crypts adjacent to either side of the wound to form a temporary protective epithelial barrier (26). We hypothesized that JAM-A effects on IEC migration are important in regulating events during such early stages of colonic mucosal repair when sheets of migratory epithelial cells are sealing eroded areas. Consistent with this hypothesis and the observed defects in mucosal wound repair, experiments conducted in vitro with 2D cultures of primary epithelial cells (colonoids) derived from murine colonoids, we observed delayed wound closure in scratch-wounded monolayers derived from *Jam-a^–/–^* and *Jam-a*^ER*Δ*IEC^ mice compared with controls. Wounded confluent epithelial monolayers of JAM-A^ERΔIEC^ colonoids displayed decreased migratory distance and velocity when compared with monolayers from *Jam-a^fl/fl^* mice, strengthening the hypothesis that JAM-A promotes epithelial migration during colonic mucosal wound repair (Figure 4).

In mechanistic studies on JAM-A function using model epithelial cell lines, we previously reported that dimerization of the extracellular domain of JAM-A triggers the formation of a PDZ-dependent signaling protein complex that includes ZO-2, the Rap activator PDZ-GEF2, and active Rap1A (9, 24). Rap1A is also known to play an important role in activation and stabilization of β1 integrin and epithelial cell–matrix adhesion. Furthermore, migration is known to be dependent on the dynamic regulation of β1 integrin containing FAs (27–30).

It is well appreciated that epithelial cell migration during wound healing requires constant and dynamic turnover of the actomyosin cytoskeleton, FA, and cell-cell adhesions (1). For these processes, spatial and coordinated activation of small GTPases, β1 integrin, and FAKs are the key regulators (31). Earlier studies reported that knockdown JAM-A in epithelial cells resulted in decreased expression of active Rap1A and integrin β1 (9). As shown in Figure 6, pulldown of active Rap1A followed by immunoblot for β1 integrin confirmed reduced levels of active Rap1 and decreased β1 integrin in JAM-A–deficient mouse IECs. Biochemical analyses for active FAKs showed decreased activity in cultured spread 2D colonoids from *Jam-a*^ER*Δ*IEC^ mice, and quantification of FAs confirmed significantly fewer and less organized punctate structures reminiscent of FAs in the leading edges of migrating epithelial cells from *Jam-a*^ER*Δ*IEC^ mice compared with control IEC. These results indicate that JAM-A deficiency in IECs in vivo results in decreased activation of Rap1, β1 integrin, and FAKs, which are key regulators of epithelial migration.

It is well recognized that Rap1 is an activator of integrins (28). Rap1 stimulation by its GEFs RAPL and RIAM activate β integrins in platelets and leukocytes, which are vital for hemostasis and leukocyte spreading and adhesion (27). Overexpression of dominant-negative Rap1A (S17A) results in defective spreading and adhesion in HeLa cells, supporting the hypothesis that Rap1A activation is essential for proper β1 integrin signaling (29). Since we observed defective epithelial spreading and FA formation in migrating 2D colonoids from JAM-A–null IECs, it is likely that this defect is secondary to decreased levels of active Rap1 and β1 integrin in JAM-A–deficient IEC.

By protein structural modeling, it was recently demonstrated that active Rap1A/Rap1B interact with the FA-associated protein Talin at the level of the F0 or F1 domain (32–35). This domain regulates the conformation of Talin to promote binding and activation of β integrins. For example, a complex formed by Rap1/Talin/β1 integrin is essential for platelet-dependent hemostasis (36). However, there are no reports of a functional role of an analogous complex in IEC. To probe whether Talin interacts with Rap1A and activates β1 integrin in IECs, and if the formation of this complex is dependent on JAM-A, we evaluated expression and localization of Talin in 2D colonoids derived from *Jam-a*^ER*Δ*IEC^ and *Jam-a^fl/fl^* mice. Immunoblot and immunofluorescence for Talin demonstrated decreased levels of Talin as well as greatly reduced localization in FA like structures in sparse cultures and at leading edges of migrating pIECs derived from *Jam-a*^ER*Δ*IEC^ mice. We also observed that talin and β1 integrin strongly coimmunoprecipitated with Rap1A in JAM-A WT IECs, but much less so in JAM-A null cells. Analyses of β1 integrin immunoprecipitates demonstrated similar results, indicating that formation of a Rap1A/Talin/β1 integrin complex is disrupted in pIEC monolayers from JAM-A–null mice. We reported that loss of epithelial JAM-A in transformed IECs decreases active Rap1, which in turn causes a decrease of β1 integrin levels. In this new report, we used pIECs to increase insights into how epithelial JAM-A regulates β1 integrin. Because Rap1 regulates Talin recruitment at FAs and Talin interacts both with active Rap1 and β1 integrin, decreased levels of active Rap1 result in reduced formation of an active Rap1/Talin/β1 integrin complex, which destabilizes β1 integrin and promotes degradation. Thus, our findings strongly support the existence of a JAM-A–dependent migratory protein complex that is essential for epithelial cell migration and wound repair.

In summary, this study demonstrates that JAM-A promotes intestinal mucosal wound healing in vivo through regulation of a promigratory protein signaling complex between Rap1A, Talin, and β1 integrin. These findings support a critical role for epithelial JAM-A in controlling mucosal repair through dynamic regulation of FAs.

## Methods

### Mice.

*Jam-a^fl/fl^* mice were generated as previously described (11). Villin^ERT2–Cre^ mice were purchased from the Jackson Laboratory (strain no. 020282). Villin^ERT2–Cre^ and Villin^ERT2–Cre^ *Jam-a^fl/fl^* mice were bred in-house at the University of Michigan. Mice were housed under specific pathogen–free conditions and used at 8–12 weeks of age. Tam treatment was conducted and previously published (14).

### DSS treatment.

Mice were provided with 3% in drinking water for times indicated (40–50 kDa, Affymetrix) as published previously (14). Clinical DAI was obtained daily, with scores of 0–4 assigned for weight loss, stool consistency, and presence of blood in stools and then divided by 3. A score of 0 represents no significant changes; 1 represents 0%–5% weight loss; 2 represents soft stools, 5%–10% weight loss, and detection of microscopic blood; 3 represents 10%–20% weight loss; and 4 represents diarrhea, macroscopic blood, and greater than 20% weight loss. Individual scores were averaged per mouse as indicated to yield stool index and DAI index scores.

### Wound healing assays.

For in vitro experiments, scratch wounding assays were performed on cell monolayers as previously described (14). Monolayers were cultured in 48-well tissue culture plates to confluency and scratched using a 10 μL pipette tip under suction. Video quantification of scratch wound closure was performed by imaging wounds at 10-minute intervals in an Axiovert Observer live-cell microscopy system (Zeiss). Wound closure was quantified at indicated time points using ImageJ software (NIH) and calculated as percent reduction of cell-free surface area compared with immediately after wounding (t = 0). Chemokinetic migration on collagen was tracked in IEC over 24 hours every 30 minutes. Motility of individual epithelial cells within clusters was tracked using ImageJ Manual Tracking and analyzed using Ibidi Chemotaxis software to obtain measures of migration including accumulated distance, Euclidean distance, and velocity. For immunoblot analysis, multiple scratches were created to enrich the migratory fraction of cells. For in vivo wounding experiments, a biopsy-based mucosal wounding model was employed as described previously (14). A high-resolution video endoscope (Coloview Veterinary Endoscope, Karl Storz) was used to create and monitor wounds in the dorsal mucosa of the descending colon of anesthetized mice (ketamine [West-Ward] at 100 mg/kg, xylazine [Akron] at 5 mg/kg).

### Intestinal enteroid and monolayer culture.

Murine small intestinal epithelial enteroids were created and maintained in culture as previously described (14). Isolated colonic crypts from untreated J*am-a*^ER*Δ*IEC^ mice were embedded in Matrigel (BD Biosciences) and maintained in L-WRN conditioned complete media supplemented with 50 ng/mL recombinant human EGF (R&D Systems). To generate JAM-A–deficient enteroid cultures, enteroids were treated for 72 hours with 1 μM (Z)-4-hydroxytamoxifen (Sigma-Aldrich) in complete media, followed by passage and maintenance in Tam-free complete media. Primary epithelial cell monolayers were generated from established colonoid cultures. Colonoids were dispersed into single-cell suspension using Trypsin/EDTA and plated in collagen I–coated 48-well tissue culture plates. Cells were cultured for 48 hours in L-WRN conditioned complete media to achieve confluency, prior to usage as indicated.

### IP and Western blotting.

For IP studies, SKCO-15 and 2D colonoids were harvested in relaxation lysis buffer (10 mM HEPES, 10 mM NaCl, 3.5 mM MgCl_2_, 100 mM KCl) with 1% Octyl-β-glucoside supplemented with a cocktail of protease and phosphatase inhibitors. Lysates were centrifuged (2,000*g*; 10 minutes; 4°C), and supernatants were collected. Supernatants were precleared with 50 μL of 50% protein A or G agarose beads (Thermo Fisher Scientific, 20333 and 20398) for 1 hour, followed by 4°C incubation with rotation overnight in the presence of 5 μg/mL antibodies or control IgG. Immune complexes were precipitated by 20 μL of protein A or G Dynabeads (Thermo Fisher Scientific, 1001D and 1003D) for 4 hours. Immunoprecipitated complexes were washed 3 times with lysis buffer before boiling in 2× NuPAGE LDS Sample Buffer (Thermo Fisher Scientific, NP0007). Immunoprecipitates and input were then subjected to SDS-PAGE followed by immunoblotting. A detailed list of antibodies used can be found in Supplemental Table 1.

### Immunofluorescence.

2D colonoids monolayers were grown on plastic chamber slides (Thermo Fisher Scientific, 177445) and fixed with either 4% paraformaldehyde (PFA) or cold 100% ethanol. PFA fixed monolayers were permeabilized with 0.5% Triton X-100 for 10 minutes. Monolayers were blocked with 3% goat or donkey serum in DPBS with 0.05% Tween-20 blocking buffer for 30 minutes. Primary antibodies were diluted in blocking buffer, and cells were incubated overnight at 4°C. Cells were washed with PBS with 0.05% Tween 20, and fluorescently labeled secondary antibodies were diluted in blocking buffer followed by incubation for 1 hour at room temperature. Cells were washed and mounted in Prolong Gold antifade agent (Thermo Fisher Scientific, P36930). Frozen ileal or colonic sections (6–8 μm) from *Jam-a^fl/fl^* and *Jam-a*^ER*Δ*IEC^ mice were fixed with 4% PFA, and immunolabeling was performed as described above. Fluorescence imaging was performed with a Nikon A1 confocal microscope (Nikon) in the Microscopy & Image Analysis Laboratory Core at University of Michigan. A detailed list of antibodies used can be found in Supplemental Table 1.

### Statistics.

Statistical significance was measured by Students’ 2-tailed *t* test or 1-way ANOVA with Bonferroni multiple-comparison correction using GraphPad Prism software. Significance was set as *P* ≤ 0.05. Averaged values are represented as the mean ± SEM.

### Study approval.

All experimental procedures involving animals were conducted in accordance with NIH guidelines and protocols approved by the IACUC at the University of Michigan.

## Author contributions

SF, KB, CKM, and MQ performed experiments in addition to data analysis/interpretation. MQ, KB, and SF wrote the manuscript. AN and CAP oversaw the project design and execution, provided assistance in writing and editing the manuscript, and acquired funding. Due to SF contributions to in vitro mechanistic cell biological studies and KB contributions to in vivo DSS and wounding experiments, we consider that SF and KB have contributed equally to this work and are co–first authors; authorship order was decided because SF was responsible to complete corrections of the paper after revision. MQ and CP are co–senior authors; order was decided based on funding.

## Supplementary Material

Supplemental data

## Version 1 08/09/2022

In-Press Preview

## Version 2 09/08/2022

Electronic publication
