# Supplementary material for: Epithelial JAM-A is fundamental for intestinal wound repair in vivo
Source: JCI Insight. 2022 Sep 8;7(17):e158934. doi: 10.1172/jci.insight.158934 (PMC9536273; doi:10.1172/jci.insight.158934)
Supplement: Supplemental data [file jciinsight-7-158934-s172.pdf]

Supplemental Figure 1

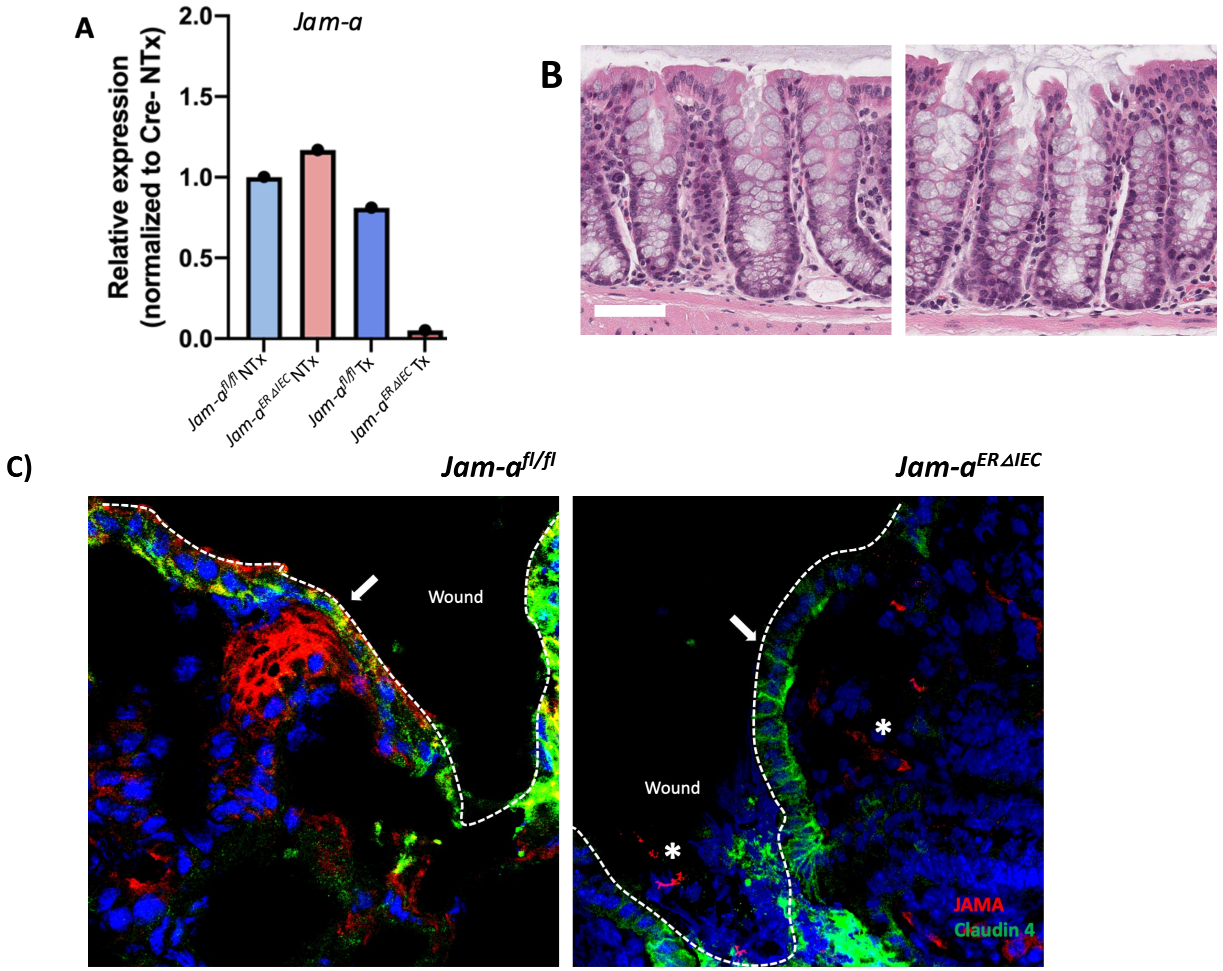


**Supplementary Figure 1. Inducible epithelial JAM-A deficient mice. A)** Jam-a mRNA relative expression showing tamoxifen treatment of *Jam-a*$^{ER\Delta IEC}$ mice leads to loss of JAM-A expression in IEC. **B)** Colonic mucosa of *Jam-a*$^{fl/fl}$ and *Jam-a*$^{ER\Delta IEC}$ tamoxifen-treated mice was embedded in Paraffin, sectioned and stained with Hematoxylin and Eosin for evaluation of histology and mucosal damage. Crypt architecture and mucosal composition are grossly normal following tamoxifen-induced epithelial loss of JAM-A (*Jam-a*$^{ER\Delta IEC}$ +Tam) when compared to Jam-a$^{fl/fl}$ Tx controls. C) Immunofluorescence showing JAM-A expression in *Jam-a*$^{fl/fl}$ and *Jam-a*$^{ER\Delta IEC}$ . Arrows indicate epithelial JAM-A expression, asterisks show lamina propria expression. Scale bar = 100 μm.

Supplemental Figure 2

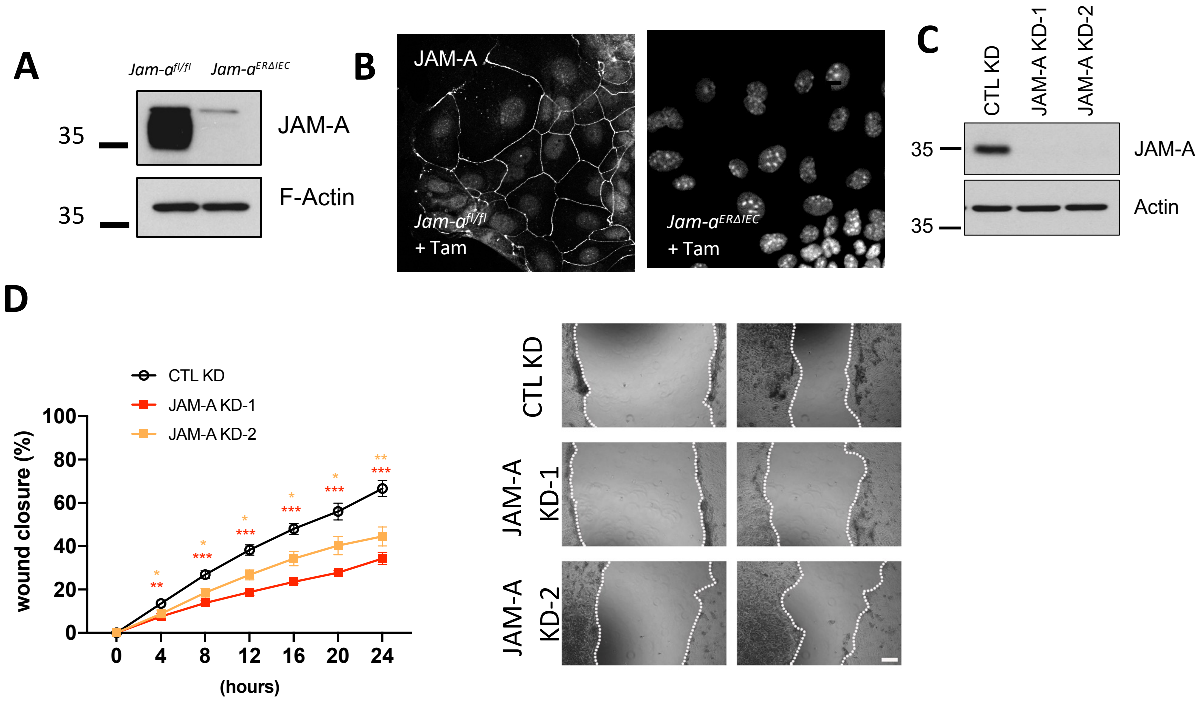


**Supplementary Figure 2. In-vitro cultures of JAM-A IEC.** Tamoxifen treatment of *Jam-a*$^{ER\Delta IEC}$ abolishes epithelial JAM-A expression in colonic mucosa **(A)** and in primary 2D cultures **(B)**. *Jam-a*$^{fl/fl}$ *derived* tissue and culture were used as controls. To corroborate in-vitro results from primary murine cells, JAM-A was knocked down in model human epithelial cells SKCO-15 **(C)**. Wound healing results are analogous to those obtained with murine primary cells where JAM-A silencing decreases wound repair **(D and E).** Data are representative of three independent experiments with at least 7 samples per group. Results are presented as means ± SEM. ****$p$ < 0.01.

Supplemental Figure 3

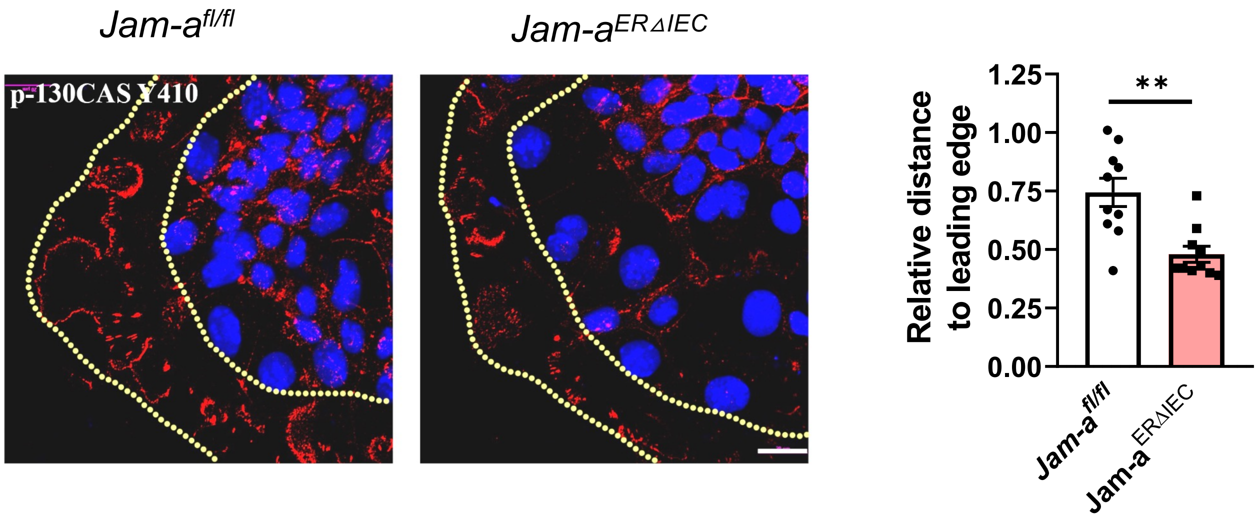


**Supplementary Fig 3. JAM-A deficiency results in IEC spreading defects**. (Left) Spread 2d colonoids were grown on chamber slides for 48 h, fixed and stained for anti-p-p130CAS Y410 with DAPI (Blue) to visualize nuclei. Scale bar = 25 μm. (Right) Quantification of distance to leading edge in spreading 2D colonoids derived from JAM-A *Jam-a*$^{fl/fl}$ and *Jam-a*$^{ER\Delta IEC}$ mice.

Supplemental Figure 4

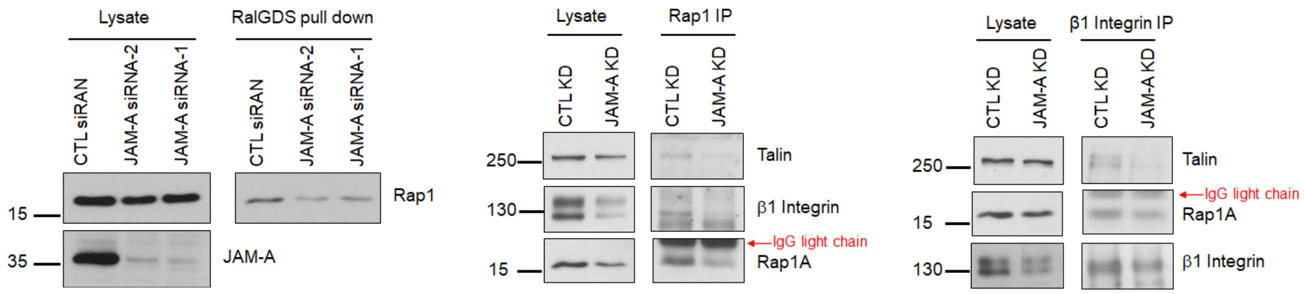


**Supplementary Fig 4.** Rap1A GTP was subjected to pull down with Rags RBD beads from lysates of spread SK CO-15 IEC. Immunoblots for Rap1A reveal less Rap1A GTP in JAM-A depleted SK CO-15 compared to control knockdown (left). Lysates from spread SK CO-15 cells after JAM-A knockdown or control knockdown were immunoprecipitated with anti-Rap1A (middle panel) or anti-β1 Integrin (right panel) antibodies followed by immunoblot for anti-talin, β1 integrin and Rap1A antibodies to evaluate composition if a functional protein complex containing Rap1A/talin/β1 integrin. Loss of JAM-A in SK CO-15 cells results in disruption of a Rap1A/talin/β1 integrin complex compared to controls.

# Supplemental Table 1

| Antibody name | Species | Company | Catalog Number |
|---|---|---|---|
| JAM-A | goat | R & D Systems | AF1077 |
| JAM-A | mouse | In-house – Clone J10.4 | N/A |
| JAM-A | rabbit | Invitrogen | 36-1700 |
| p130CAS | rabbit | Cell Signaling Technology | 13846 |
| p-p130CAS Y410 | rabbit | Cell Signaling Technology | 4011 |
| Talin | mouse | Sigma-Aldrich | T3287 |
| β1 Integrin | rabbit | Millipore | AB1952 |
| β1 Integrin | rat | R & D system | MAB2405 |
| Paxillin | rabbit | Abcam | Ab32048 |
| FAK | rabbit | Cell Signaling Technology | Rap1A |
| p-FAK Y861 | rabbit | Millipore | PS1008 |
| Rap1A | rabbit | ThermoFisher | PA5-21457 |
| Rap1 | rabbit | Millipore | 07-916 |
| Src | rabbit | Cell Signaling Technology | 2123 |
| p-Src Y416 | rabbit | Cell Signaling Technology | 6843 |
| Calnexin | rabbit | Sigma-Aldrich | C4731 |
| β-Actin | mouse | Millipore | T6557 |
